# Editorial: Precision nutrition and dietary interventions in the management of metabolic dysfunction-associated steatotic liver disease (MASLD)

**DOI:** 10.3389/fnut.2026.1924585

**Published:** 2026-07-29

**Authors:** Berenice M. Román-Calleja, Bryan Adrian Priego-Parra, Carlos Fernando Martínez-Cabrera

**Affiliations:** 1 Hepatology and Liver Transplant Division, Instituto Nacional de Ciencias Médicas y Nutrición Salvador Zubirán, Mexico City, Mexico; 2 Laboratorio de Fisiologia Digestiva y Motilidad Gastrointestinal, Universidad Veracruzana, Veracruz, Mexico; 3 Centro de Investigación y Gastroenterología, Mexico City, Mexico

**Keywords:** dietary interventions, MASLD, metabolic biomarkers, nutrition precision, nutritional assessment

## Introduction

Metabolic dysfunction-associated steatotic liver disease (MASLD) is the most common chronic liver disease worldwide, affecting nearly one-third of the global population (1). Lifestyle modification, including nutritional intervention, remains the cornerstone of MASLD management. Although weight loss of >7%−10% is recommended to improve liver histology and metabolic outcomes, individual responses to dietary interventions vary considerably, reflecting the heterogeneous clinical, metabolic, and biological characteristics of the disease.

**Figure 1:**
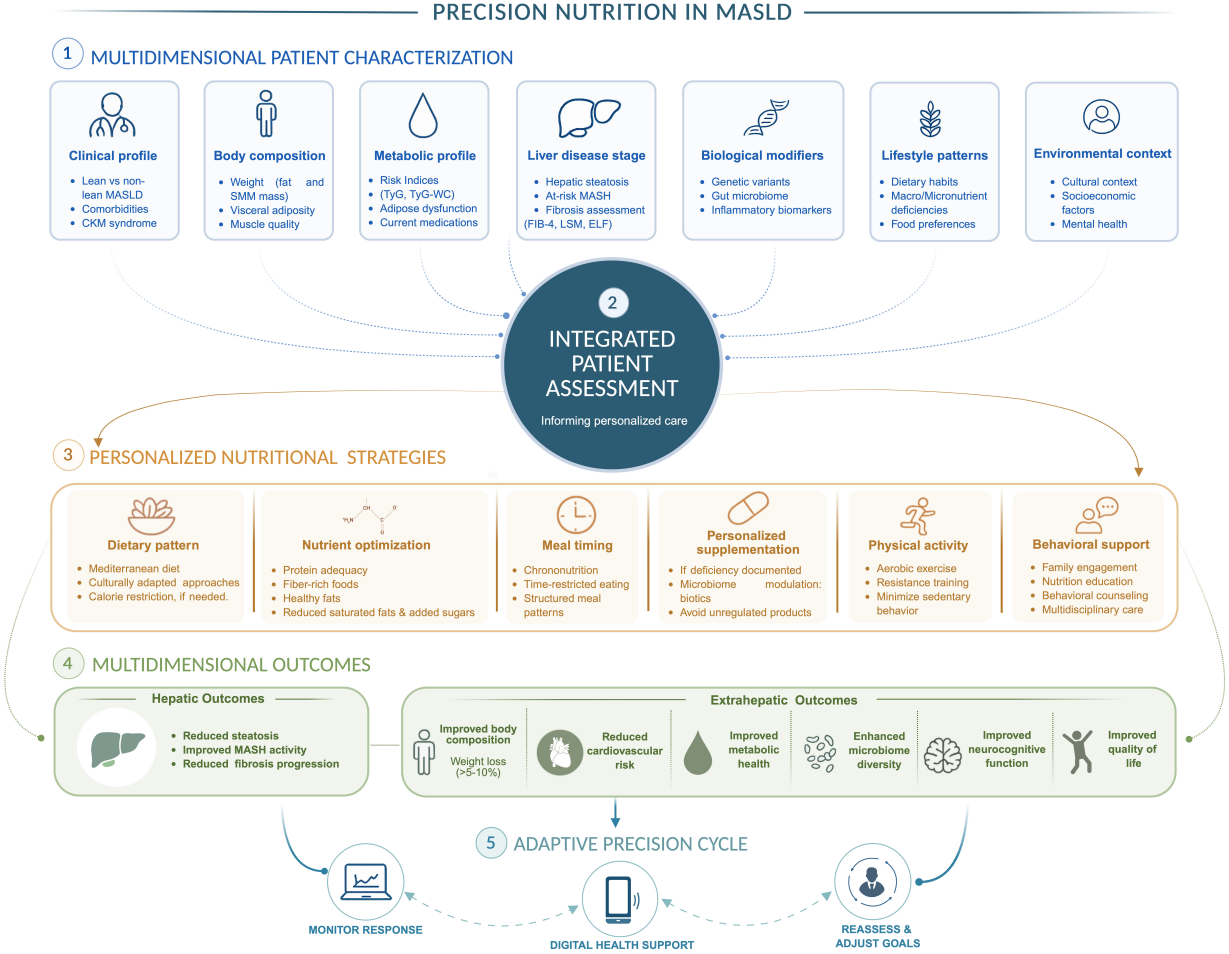
Conceptual framework of precision nutrition in the management of metabolic dysfunction-associated steatotic liver disease (MASLD). The articles included in this Research Topic contribute to different components of this conceptual framework, collectively illustrating how precision nutrition can be translated into clinical practice.

Precision nutrition has emerged as a promising framework to address this heterogeneity by integrating lifestyle and behavioral factors, body composition, metabolic biomarkers, environmental exposures, cultural preferences, and genomic, molecular, and microbiome-related characteristics into individualized nutritional approaches (2). As illustrated in Figure 1, precision nutrition in MASLD encompasses a continuum from multidimensional patient characterization and integrated patient assessment to the implementation of personalized dietary and lifestyle interventions aimed at improving both hepatic and extrahepatic outcomes.

The articles assembled in this Research Topic illustrate how the principles of precision nutrition can be translated into practical strategies for the management of MASLD.

## Integrated patient assessment as a cornerstone of precision nutrition

The marked heterogeneity partly explains why uniform nutritional recommendations often produce variable responses in MASLD. Integrated patient assessment provides a framework to identify clinically meaningful sources of heterogeneity that may influence disease progression and response to dietary interventions. Metabolic and inflammatory phenotyping are key components of this multidimensional assessment. In this context, Ntikoudi emphasizes that clinically meaningful metabolic and inflammatory profiles can be identified using readily available biomarkers, such as HOMA-IR, TG/HDL-C, and hs-CRP, without necessarily relying on complex omics technologies.

Along similar lines, Priego-Parra et al. (3) highlighted the triglyceride-glucose-waist circumference (TyG-WC) index as a practical surrogate of insulin resistance, visceral adiposity, and cardiometabolic risk, illustrating how accessible metabolic biomarkers can guide personalized nutritional management in MASLD. These advances reinforce the growing role of simple metabolic and nutritional biomarkers in refining patient assessment and guiding precision nutrition.

## Metabolic and nutritional biomarkers in precision nutrition

Accessible metabolic and nutritional biomarkers are emerging as key tools for refining integrated patient assessment and improving risk stratification in MASLD. In this context, Cheng and Wang demonstrated that the single-point insulin sensitivity estimator (SPISE), a surrogate marker of insulin sensitivity derived from routinely available clinical parameters, was inversely associated with both prevalent and incident MASLD. Similarly, Xuan et al. identified the fasting blood glucose-to-high-density lipoprotein cholesterol ratio as another accessible metabolic biomarker associated with MASLD risk. Complementing these findings, Song et al. showed that several non-conventional lipid-derived indices, particularly TyG-BMI, performed excellently in identifying individuals with steatotic liver disease, further supporting the use of simple, readily available biomarkers in metabolic risk assessment. Expanding beyond insulin resistance-related indices, He et al. identified a U-shaped association between plasma homocysteine and incident MASLD, with both low and high concentrations associated with increased risk, suggesting that one-carbon metabolism may represent an additional metabolic pathway contributing to disease susceptibility.

Nutritional status also represents an important dimension of integrated patient assessment. Guo et al. demonstrated that higher geriatric nutritional risk index (GNRI) values were consistently associated with an increased risk across major MASLD subtypes, including diabetes-related, overweight/obesity-related, and lean MASLD, in older adults.

## Dietary patterns and nutritional exposures

Beyond its role as a relevant therapeutic intervention, diet has been recognized as a significant contributor to the development of MASLD and a major modifiable risk factor. In this context, Qiu et al. showed that greater adherence to the Planetary Health Diet Index (PHDI) was associated with a lower likelihood of MASLD, with the effect partly mediated by body mass index and waist circumference. In addition, Yang et al. demonstrated that greater adherence to the MIND Diet Score was associated with improved hepatic and metabolic health together with a more favorable gut microbiota profile, supporting the potential role of comprehensive dietary quality indices in precision nutrition. Siani et al. further reported an inverse, dose-dependent association between Italian-style coffee consumption and MASLD risk, with lower odds observed as daily coffee intake increased. Although this observational study does not establish causality, it highlights the potential relevance of specific dietary exposures in modulating MASLD risk. Taken together, these studies reinforce the importance of dietary quality and selected nutritional exposures in MASLD and support the inclusion of both overall dietary patterns and individual dietary components in nutritional assessment.

## Mechanistic insights supporting targeted interventions and future directions

Dietary interventions, reduced intake of sugar-sweetened beverages (Li et al.), and regular physical activity improve metabolic health and body composition while modulating key pathways involved in disease progression, including insulin resistance, systemic inflammation, and gut microbial ecology. Growing evidence positions the gut microbiota as a key mediator linking environmental exposures with hepatic and metabolic dysfunction. Within this framework, diet should be considered not only as a source of nutrients but also as a therapeutic modulator of host–microbiome interactions. Bioactive components, including omega-3 fatty acids, coffee, tea, and fermented foods, have been associated with favorable metabolic and microbial profiles, although their molecular mechanisms remain incompletely understood.

These effects are closely related to the gut–liver axis, through which microbial metabolites and microbial-associated molecular patterns influence hepatic homeostasis. During dysbiosis, increased exposure to lipopolysaccharides, altered bile acid metabolism, and other microbial products may promote adipose tissue dysfunction, oxidative stress, mitochondrial impairment, and chronic inflammation, thereby supporting dietary and microbiota-targeted approaches as therapeutic strategies (4).

Pharmacological therapies complement lifestyle interventions by targeting metabolic, inflammatory, fibrotic, and immune-related pathways, including PPAR, GLP-1 receptor, SGLT2, FXR, DPP-4, FGF, and related signaling pathways (5). Additionally, some supplements, such as carnitine, have shown therapeutic potential, as highlighted by Chen C. et al.

Beyond current therapies, NLR inflammasome activation has emerged as a potential mechanistic link between dysbiosis and metabolic inflammation, supporting future strategies to modulate immune-metabolic pathways (6).

Translating these advances into clinical practice will require a precision-medicine framework that goes beyond conventional disease classification. Metabolic and inflammatory phenotyping may identify biologically distinct subgroups and guide targeted interventions. Chen H. et al. further showed that intermittent fasting may exert metabolic and gene-expression effects beyond those of caloric restriction alone.

Collectively, these advances support a shift from a liver-centered view of MASLD toward a systems-based framework integrating host metabolism, immunity, and the intestinal microbiome. This perspective aligns with the concept of the human holobiont and may facilitate the development of more precise and biologically informed therapeutic strategies.

## Conclusions

Lifestyle modification remains the cornerstone of MASLD management, with dietary intervention and increased physical activity representing the foundation of current therapeutic strategies. However, substantial interindividual variability in treatment response highlights the limitations of a uniform approach. Integrating precision nutrition into clinical care offers an opportunity to tailor dietary interventions according to each patient's metabolic phenotype, body composition, comorbidities, lifestyle, and, where appropriate, multi-omics data. Such an individualized approach has the potential to improve adherence, maximize metabolic and hepatic benefits, and ultimately enhance long-term disease management.

## References

[1] Tilg H Petta S Stefan N Targher G. Metabolic dysfunction-associated steatotic liver disease in adults: a review. JAMA. (2026) 335:163–74. doi: 10.1001/jama.2025.19615 41212550

[2] da Silva BR Brennan L Horst MA Wishart DS Prado CM. Advancing precision nutrition: bridging mechanistic insight and clinical implementation. Nat Rev Endocrinol. (2025) 21:515–7. doi: 10.1038/s41574-025-01141-9 40523984

[3] Priego-Parra BA Román-Calleja BM Gallego-Duran R Gracia-Sancho J Velarde Ruiz-Velasco JA Remes-Troche JM. Triglyceride-glucose-waist circumference index: a powerful tool for metabolic dysfunction-associated steatotic liver disease. World J Hepatol. (2025) 17:1–15. doi: 10.4254/wjh.v17.i7.107668 40747214 PMC12308583

[4] Acierno C Caturano A Barletta F Rinaldi L Sasso FC Adinolfi LE . Nutritional interventions targeting the gut microbiome in MASLD: from prebiotics and probiotics to postbiotics and fecal microbiota transplantation. Nutrients. (2026) 18:1765. doi: 10.3390/nu18111765 42280407 PMC13258734

[5] Mejía-Guzmán JE Belmont-Hernández RA Chávez-Tapia NC Uribe M Nuño-Lámbarri N. Metabolic-dysfunction-associated steatotic liver disease: molecular mechanisms, clinical implications, and emerging therapeutic strategies. Int J Mol Sci. (2025) 26:2959. doi: 10.3390/ijms26072959 40243565 PMC11988898

[6] Zhou J Yang R Zhu W He Z Ma Y Feng Z . NLR inflammasome pathways: key targets for pathogenesis and therapy of metabolic diseases. Front Immunol. (2026) 17:1823724. doi: 10.3389/fimmu.2026.1823724 42292355 PMC13257947

